# The Current State of Artificial Intelligence in Medical Imaging and Nuclear Medicine

**DOI:** 10.1259/bjro.20190037

**Published:** 2019-10-16

**Authors:** Louise I T Lee, Senthooran Kanthasamy, Radha S Ayyalaraju, Rakesh Ganatra

**Affiliations:** 1 Department of Radiology, University Hospitals of Leicester, Leicester Royal Infirmary, England, UK; 2 Department of Trauma and Orthopaedics, Cambridge University Hospitals, Cambridge University Hospitals, Addenbrooke’s Hospital, England, UK; 3 Department of Dermatology, Queens Medical Cente,Nottingham, England, UK; 4 Department of Nuclear Medicine, University Hospitals of Leicester, Glenfield Hospital, England, UK

## Abstract

The last decade has seen a huge surge in interest surrounding artificial intelligence (AI). AI has been around since the 1950s, although technological limitations in the early days meant performance was initially inferior compared to humans.^1^ With rapid progression of algorithm design, growth of vast digital datasets and development of powerful computing power, AI now has the capability to outperform humans. Consequently, the integration of AI into the modern world is skyrocketing.

This review article will give an overview of the use of AI in the modern world and discuss current and potential uses in healthcare, with a particular focus on its applications and likely impact in medical imaging. We will discuss the consequences and challenges of AI integration into healthcare.

## Introduction

It is important to first and foremost define artificial intelligence (AI), as well as other commonly used descriptive terms linked with AI such as machine learning and deep learning.

AI is an area of computer science that involves the creation of machines capable of performing like humans and perceiving the world as we do.^2^

Machine learning is a form of AI concerning the development of computer programmes that can find patterns within complex data sets and produce intellectual predictions without explicit human programming. Experience is gained through exposure to more data, with systems capable of excellent problem solving skills and the potential to extract information from data that humans cannot.^3^

Three types of machine learning exist: supervised, unsupervised and reinforcement learning. Supervised training teaches the model a labelled input data set that is associated with the correct output. Unsupervised training involves detecting patterns within data in an unknown outcome, thereby training itself on data.^4^ Reinforcement training explores each action in turn and discovers which of these results in reward by receiving positive or negative reinforcement, thus learning from consequences of interactions.^5^

Numerous machine learning applications have been made possible by breakthroughs in deep learning. Deep learning is a branch of machine learning where algorithms are structured into numerous processing layers based on an artificial neural network inspired by the human brain. These structures learn representations of data with multiple levels of abstraction and have the ability to link complicated nonlinear relationships and predictions without human intervention.^6^ The convolutional neural network (CNN) is a deep learning model which is used most widely in medical imaging.^7^

AI already forms part of our every day lives. Deep learning has been incorporated into numerous products created by many of the world’s largest technological giants including Apple, Google, Microsoft and Facebook.^8^ Applications are capable of facial recognition, speech recognition, language translation, web searches and autonomous driving to name but a few.^9^ Not only has AI stormed the technology industry, there has been widespread use in the financial, banking, marketing and manufacturing sectors.^6,10^ Whilst AI has been widely used in other fields, it has only recently gained momentum in medical application.

## Medical applications of AI

### Computer-assisted detection

One of the first uses of AI in the healthcare sector was the introduction of computer-assisted detection (CAD) in the 1980s. CAD is a form of AI utilizing pattern recognition to identify and highlight areas of concern on an image. Highlighted areas are flagged to the user for review.^11^ Although a form of AI, it is not a type of machine or deep learning as the system relies on rule-based algorithms.^10^

CAD gained Food and Drugs Administration (FDA) and CE approval in 1998 for use in screening and diagnostic mammography, as well as in plain chest radiography and CT chest imaging. For clinical implementation, the FDA requires the user to first review and interpret images, then CAD marks are displayed to highlight areas for re-review. The idea behind the technology is to decrease observational oversights and ultimately false-negative rates in disease detection.^12^

#### CAD in mammography

CAD is useful in screening and diagnostic mammography for the detection and demarcation of microcalcifications, lesions and architectural distortion.^13^ The system essentially acts as a second opinion to the radiologist’s first read. Overall evidence for the added clinical value of CAD in mammography remains equivocal. Some studies have concluded use of CAD increases the detection of cancer with a small rise in recall rate.^14^ Conversely, Taylor et al conducted a systematic review that analyzed 27 studies and concluded CAD increased recall rates and flagged a relative large number of false-positive images, but there was a lack of evidence of overall improved cancer detection rates.^15^ At present, the USA routinely uses CAD in breast screening programmes, whilst the UK does not.^11^

#### CAD in chest radiology

CAD has been used in chest radiology for lung nodule detection in both plain film and CT with focus on early detection of lung cancer.^16,17^ Up to 19% of nodules on chest radiographs are missed and up to 38% on CT due to observational oversights.^18,19^ Sahiner et al looked at nodules missed by radiologists and found that CAD detected 56–76% of these lesions. The study concluded CAD had the potential for detecting early stage one lung cancer.^16^ Other studies have assessed the use of CAD in detecting other lung pathologies including emphysema, pleural effusion, pneumonia, pneumothorax and interstitial lung disease. These have produced variable outcomes.^20^

### Deep learning CNNs

Unlike deep learning, traditional CAD systems require training before use by pre-taught algorithms. AI research has evolved such that systems now incorporate deep learning CNNs to integrate and build on new information without reprogramming from humans. Development of such technology has greatly increased their usefulness within the medical imaging field.^21^ Deep learning algorithms are particularly successful in highly technical medical specialties that rely on imaging such as histopathology, dermatology, ophthalmology and radiology.^22^

#### Histopathology

A central component of staging of breast cancer involves the microscopic examination of lymph nodes for metastasis following nodal clearance. Detection of disease in nodes is both time consuming and error prone, particularly for small tumours. Liu et al evaluated the application of deep learning-based AI for the detection of metastatic breast cancer in sentinel lymph node biopsies using Lymph Node Assistant (LYNA). LYNA achieved high tumour level sensitivity compared to pathologists, with the study suggesting the technology could be used to improve the accuracy of detecting tumour cells. Furthermore, the technology could be used to augment pathologists’ workflow by automatically flagging challenging slides or negative slides, or for flagging slides for a second read in potentially missed metastasis.^23^

#### Dermatology

Esteva et al demonstrated the effectiveness of deep learning in dermatology. Deep CNN systems classified skin lesions trained on a dataset of 129,450 clinical images containing 2032 skin diseases. Performance of the system was tested against 21 board-certified dermatologists with two binary classifications: keratinocyte carcinomas versus benign seborrhoeic keratosis and malignant melanomas versus benign naevi. Their system classified skin cancer with impressive accuracy, matching the performance of the dermatologists across three diagnostic tasks: keratinocyte carcinoma classification, melanoma classification and melanoma classification using dermatoscopy.^24^

#### Ophthalmology

Gulshan et al applied deep learning algorithms to detect diabetic retinopathy and diabetic macular oedema in retinal fundus photographs. The performance of the deep learning algorithm was compared with manual grading by ophthalmologists. Trained on a dataset of 12,8175 retinal images, the deep CNN had high sensitivity and specificity for detection of referable diabetic retinopathy.^25^ AI use in ophthalmology expands to detection of diseases with high incidence such as age-related macular degeneration, glaucoma, retinopathy of prematurity and age-related/congenital cataracts.^26^

## AI in radiology

### Workforce issues

It is well known that the UK radiology workforce has been in crisis for a number of years. This has placed a huge strain on radiology departments and on the workers. AI has the potential to transform the workflow of radiologists, carrying a positive knock-on effect for departmental efficiency as well as improved patient outcomes.^27^

The Royal College of Radiologists (RCR) highlighted key issues in the 2017 workforce census report. The vast majority of patients undergo some form of imaging during hospital admission. Improvement in imaging techniques and the wider availability of imaging has resulted in an estimated increase of 30% in reporting workload over the past 5 years. The increase in demand has been particularly prevalent in more time consuming imaging such as CT and MRI. Radiologists are also challenged with reporting a larger volume of acute imaging in a timely manner.^28^

An estimated shortfall of 1000 consultants in the UK in 2017 is projected to rise to 1600 over the next 5 years. Although there has been an increase in the number of radiology trainees to try to meet this shortfall, more needs to be done and alternative strategies have to be considered. 97% of radiology departments are failing to meet their reporting requirements within staff contracted hours. Many departments are relying on outsourcing and insourcing in an attempt to deal with the increasing demands. Despite this, numerous scans remain unreported, leading to delayed or missed diagnoses and an adverse impact on patient care.^28^

### Suitability of radiology for AI integration

AI in healthcare aims to improve early diagnosis and optimize patient management. A myriad of AI projects with focus on radiology have been developed and research is progressing at a rapid pace.

Success of deep learning systems relies heavily on the availability of massive electronic datasets known as “bigdata.” Naturally, radiology is a specialty particularly suited to such integration, as the specialty already has a large digital data set. Most hospitals use electronic healthcare databases such as Picture Archiving and Communications System for reviewing and organizing images, the Radiological Information System (RIS) for managing medical imaging and associated data, whilst Electronic Medical Records (EMR) collates clinical data including notes, pathology and laboratory data.^29^

### Is AI a threat to our jobs?

In order to understand how AI might shape future radiology practice, it is important for radiologists to understand what scope the technology has to redefine their role and augment their skillset. Understandably, the hype surrounding AI has lead to fears that machines will replace radiologists in the future. Radiology as a specialty needs to embrace the changes that AI promises to make possible. Rather than being replaced by machines, AI holds the key in complementing the skills unique to the radiologist, whilst solving many workforce issues within the specialty. The role of radiologists will invariably continue to expand as the demand for imaging increases and technological advances in imaging modalities improve.

AI will allow radiologists to expand their other roles including communication with clinicians, leading interventional procedures and conducting quality assurance and quality improvement projects.^30^ Radiologists have the unique role of integrating information from imaging to clinical data, offering valuable insight to other medical professionals in the diagnosis and individualized management of patients. Machines do not have the ability to engage in such complex conversations with humans, nor do machines have the empathy capable of matching a human doctor and this remains a fundamental aspect of a doctor’s role.^31^ Radiologists have, and will continue to hold an influential and irreplaceable role in patient care, while AI can help enhance such a position.

### Automation of reporting

Areas where AI can contribute include automation of standardized radiology reports, image segmentation, lesion measurement and comparison of scans with previous imaging.^32^ Radiologists report vast numbers of follow-up studies for indeterminate findings, for monitoring treatment response or in disease relapse. These involve laborious, time-consuming tasks such as lesion measurement and describing morphology. Such tasks may benefit from automation using AI hence freeing up radiologists to focus on other complex tasks.^33^

Natural language processing of radiology reports also poses the potential of generating reports altered to suit the reader, be it the patient, specialty doctor or primary care doctor. There is also the potential of deep learning algorithms to insert automated recommendations for the clinician at the end of the report when critical findings are detected on a scan.^29^ Furthermore, research is looking into developing a system that can track radiologists’ recommendation, ensuring patients are not lost to appropriate follow-up.^34^

### AI in nuclear medicine

The scope of automated image interpretation extends to functional imaging. Quantification and comparison to normal data sets has existed in myocardial perfusion imaging for decades and machines can already produce “difference maps.“ Kim et al demonstrated the use of deep learning in the automated diagnosis of Parkinson’s disease from dopamine active transporter (DAT) single photon emission CT (SPECT) scans, achieving a sensitivity of 96%.^35^ Choi et al also developed a deep learning-based system that accurately interpreted SPECT scans for Parkinson’s disease, achieving a sensitivity of 94% and overcame human interobserver variability.^36^ Applications can also be extended to PET-CT and PET-MRI. As technology progresses, AI will become more capable of handling the larger data sets of hybrid functional and structural imaging such as SPECT and PET images.

### Use beyond image interpretation

The scope of AI use in radiology extends well beyond automated image interpretation and reporting. Much research has focussed on optimizing workflow and improving efficiency on the whole.

The UK has seen a 30% increase in imaging demand over the past 5 years. It is essential that a system is in place capable of prioritizing reporting work lists.^37^ Reporting can be classified into non-urgent, urgent or critical. Scans with time-critical findings which impact on patient management can be highlighted to the radiologist for reporting first, whilst scans with negative findings can be deprioritized. Reshuffling of work lists mean critical findings can be relayed to clinicians more quickly and efficiently, with resultant positive impact on patient outcome. Furthermore, if AI accurately filtered out normal scans, the radiologist could spend more time on the more complex cases.

A key aspect of interpreting imaging involves producing a report that is of added clinical value to the clinician in order to help with patient management. Radiologists often do not have easy access to important and relevant patient information such as laboratory results, histopathology results and clinical notes that would help shape conclusions of reports. The introduction of EMR has meant patient data can be pooled onto one system from various sources. AI holds the potential of extracting pertinent information from such sources to allow radiologists access to key information for reporting.^29^

Since its introduction, CT has evolved as an important imaging modality which is now used 24/7 in hospitals. However, one of its biggest drawbacks is radiation dose. Reducing the radiation dose would make its use safer, although doing so would create noisier images, which are more challenging to interpret accurately. Deep learning techniques are now able to map what structures look like in low dose imaging compared to regular dose imaging, generating diagnostic quality comparable to regular CT imaging.^38–40^

Whilst MRI does not have the same radiation issues, one of the biggest limitations is the relatively long acquisition time. Chaudhari et al used deep learning to reduce acquisition time by improving image quality of thicker MR sections, comparable to that of thin sections by interpolating data.^41^ Furthermore, Hyun et al achieved faster MRI by presenting a deep learning method that used 29% of k-space data to generate images comparable to standard MRI reconstruction with fully sampled data.^42^

### RCR framework for AI implementation

Recognizing that AI has the potential to transform medical care, the RCR and British Institute of Radiology welcomes the use of AI to positively enhance clinical practice. The future sees AI integration into the daily workflow of radiologists, with hopes of improving efficiency and the radiologist’s diagnostic capacity, freeing up more time for direct patient care and Research and Development activities. AI will allow radiologists to play a more effective diagnostic role by enabling data analysis from various sources, rather than just from image-based data.^27,43^

The success of deep learning technology relies on the availability of and access to vast volumes of electronic data sets from which learning can occur. The RCR has welcomed the government’s challenge to the NHS, AI sector and health charities to use “bigdata” and AI to revolutionize the diagnosis and treatment of chronic disease. The government has ambitious plans of decreasing death from cancer by around 22,000 each year by 2033.^44^ For this to be possible, it is necessary to access NHS “bigdata” in a manner that is secure, safe, legal and anonymized, and to enable a strategy for non-commercial, robust testing of AI before use on patients to ensure safety and accuracy of such technology. As yet, there is no regulatory framework in place for AI integration into our healthcare system.^27^ There is a need for appropriately regulated and governed use of AI technology to ensure both medical professionals and patients alike gain confidence in the technology.

### AI projects across the UK

A recent symposium in May 2018 conceived by the RCR, in partnership with The Alan Turing Institute, Health Data Research UK and Engineering and Physical Sciences Research council considered the grand challenges of AI and discussed current projects in development across the UK (Table 1).^45^

**Table 1.** Current projects in development across the UK

Location	Brief description of projects
Oxford	“Optellum”—software provides an objectively determined risk score of lung nodule malignancy. So far has detected nodules on CT images with almost 100% accuracy. “Auto-prognosis” programme—100 machine learning programmes are being fused to identify risk factors for breast cancer and determine individualized optimal breast imaging techniques for those undergoing screening.
Imperial College London	“Machine learning in whole body oncology”—automatic detection and segmentation of lesions from whole body MRI scans to aid in cancer staging. Another study is combining patients’ MRI determined heart function to long-term prognostic data to determine individual risk of developing conditions such as pulmonary hypertension.
Manchester	A screening tool is being developed to predict breast cancer in patients by identifying high-risk patients suitable for early intervention and extra screening.

### Companies developing AI for healthcare

Industry has already recognized the potential of AI in healthcare. Table 2 highlights a few companies to provide an idea of the scale of the rapid expansion of AI over the past decade.

**Table 2.** AI companies with brief description of AI development

Company	Brief description of AI development
IBM Watson	Supercomputer developed by IBM. 30 billion anonymized medical images are available for Watson to train on since IBM acquired Merge Healthcare. “Eyes of Watson” has been showcased at the RSNA in 2016. Attendees experienced interactive demonstrations in cardiology and mammography, highlighting the potential in assisting radiologists.^46,47^
DeepMind	London-based company. DeepMind has collaborated with hospitals in the London: Moorfields Eye Hospital—to analyze eye scans, looking for signs that may lead to blindness; University College London Hospital—to develop algorithms capable of diagnosing head and neck cancer on CT and MRI scans; Imperial College London—to improve breast cancer detection on mammography and Royal Free Hospital London—to develop clinical mobile applications linked to EMR to help with acute kidney injury management.^48–51^
MaxQ-AI	Israel-based company. Developed software capable of detecting ICH on CT. Recently partnered with IBM Watson to integrate their ICH detector for use in Emergency Departments. Also teamed up with Samsung for use in mobile stroke units, allowing clot-dissolving drugs used in ischaemic stokes to be administered en route to hospital.^52,53^
Enlitic	San Francisco-based company. Developed algorithms capable of increasing accuracy of radiology report interpretation by 50–70% at a speed 70,000 times faster. Aims to improve radiologists’ workflow by improving ability to identify and characterize abnormalities.^54^
Viz.ai	San Francisco-based company. Developed first computer-aided triage software to analyze CT scans for stroke. Notifies specialists if a large ischaemic stroke is identified, helping to decrease time to critical treatment. Notifications save an average of 52 min in >95% of cases.^55^

AI, artificial intelligence; ICH, intracranial haemorrhage; RSNA, Radiological Society of North America.

## Challenges of implementation in Medical Imaging

Implementation of AI for use in daily clinical practice will not be without significant challenges that need to first be tackled, in order to see a smooth transition into a future with AI.

### Anonymization of sensitive data

Success of deep learning algorithms largely depend on the availability of massive data sets to allow training. Although most patient data are now available in electronic format, data availability is simply not enough. Sensitive data need to be accessed in a safe and anonymized way. AI systems should be able to learn and build on information from patients without needing personally identifiable information.^27^ Visions of a National NHS-fed imaging database, so called the BRAIN have been initiated. British Radiology AI Network would potentially be a national host of anonymized NHS imaging data across the UK, with granted access to AI developers wishing to train AI models. Another challenge would be creating a universally standardized image type across hospitals Picture Archiving and Communications System to produce a national pool of data accessible by developers.^45^

### Data labelling

Success of AI depends not only on the availability of large data sets for training, but also on accurate data labelling. This needs to be performed by adequately trained readers to ensure accuracy and credibility. Inaccurate data labelling would carry profound impact on the computer’s learning process. Subsequent learning would be erroneous, decreasing the overall accuracy of the machine. Furthermore, mislabelling a cancer as normal would not only impact the learning process, but would also carry a potentially fatal impact on patients.

### Generalization into clinical practice

Data protection laws vary internationally. Some geographical locations implement legal restrictions to protect data from leaving physical locations. This poses a significant limitation for researchers who use deep learning algorithms. Lack of generalization of training poses a limit for translation into clinical practice by healthcare providers across the world.^56^ Clinical practice using AI requires an interconnected network of patient data sets so that AI is both robust and generalized across various patient demographics, disease and regions around the world.^57^

Rare diseases may cause limitations for the accuracy of deep learning algorithms, as there will be a relatively small amount of data for algorithms to train on. Similarly, algorithms that predict outcomes from genetic findings may lack generalizability if there are only a limited number of studies in certain populations.^58^

Normal anatomical variants also pose a risk of lack of generalizability. The human body has great variation in normal dimensions of structures and textures, such that variations can potentially mask pathological conditions. Machines could find it a challenge to learn normal variants (particularly in cases of limited databases) and disregard such variations as normal rather than disease, especially if these are rare.^33,58^

### Regulation

There is currently no official regulatory framework for AI implementation into clinical use. AI systems need to be rigorously tested and results published on sensitivity and specificity before it is adopted into clinical practice, to ensure the health of patients is safeguarded.^27^

AI technology developed by private sector designers carry the risk of unethical intentions when creating technology for clinical use. Hypothetically, users of AI technology may be guided to managing patients that could generate a higher profit margin for the developer, but not necessarily translate to better patient care. Examples include using recommended drugs or medical devices in which private sector designers are stakeholders.^58^ For these reasons, appropriate regulation is ever more important, especially in complex cases where optimal management is not standardized and debated even amongst clinicians.

In the USA, the FDA regulates use of medical devices. The rapid development of AI and the “black box” nature of such devices make it difficult for the FDA to approve such devices in a timely fashion. For example, CAD, a system based on pre-taught algorithms took many years before the FDA granted approval for clinical use. Deep learning technology systems that do not require supervision may be even more difficult for the FDA to approve.^58^

### Accountability

A big question posed by integration of AI into healthcare is accountability. If an AI system making autonomous decisions about patient management fails, leading to patient harm, who is held accountable? There has been much discussion regarding the “black box” nature of AI technology. Machines have exhibited impressive results, capable of self-taught learning and the ability to comprehend scenarios more quickly and precisely compared to humans. However, there is a lack of understanding of complex algorithms. Developers of such technology are at times unable to understand and explain how their technology has arrived at a certain result.^31^ If an AI system was to fail, would this mean the developer is accountable even though the unanticipated has occurred, or is it the fault of the clinician who would be relaying these results to the patient? Furthermore, if clinicians are unable to explain how a piece of technology has arrived at a certain conclusion, patients may not have confidence in accepting results drawn by AI.

With this in mind, the Defence Advanced Research Projects Agency has developed a programme named Explainable AI to develop systems that have the ability of explaining how conclusions have been drawn from scenarios and to improve overall understanding of deep learning technology.^59^

### Susceptibility to cyber attacks

Implementation of AI systems means access to sensitive health data. Access to sensitive information always carries the risk of cyber attacks, posing a substantial risk on patient privacy. Recently, there has been a growing concern over privacy and personal data regulation. There is a fine balance between privacy and enhanced user experience when using personal data.^58^

Recognizing this, the UK government has implemented the EU directive on the Security of Networks and Information Systems (NIS Directive) to protect the UK in cyber space, as part of the £1.9 billion National Cyber Security Strategy.^60^

## Conclusion

The technological era has truly arrived. It forms an integral part of our everyday lives and most of us would be lost without our phones and laptops. AI is simply an extension of this and has the potential to significantly impact on healthcare. It is already being trialled in Medical Imaging and has shown significant advance in chest and breast radiology with scope to utilize it in all modalities including functional imaging. There is scepticism about its accuracy and challenges it faces, as well as an understandable fear of it replacing radiologists. However, it offers scope to revolutionisze the practice of radiology, making it safer, more clinically appropriate and issuing reports in a more user friendly and timely manner. The technology will develop with or without the involvement of Radiologists. Radiologists need to embrace the technology and help develop it to fulfil appropriate clinical need, as well as adapt their daily practice to ensure AI works with them rather than being a threat to their roles. Appropriate acceptance and involvement will ensure optimal technological development to improve our working lives and better serve the health service.
